# A Comparison of Polysaccharide Substrates and Reducing Sugar Methods for the Measurement of *endo*-1,4-β-Xylanase

**DOI:** 10.1007/s12010-015-1803-z

**Published:** 2015-08-20

**Authors:** Barry V. McCleary, Paraic McGeough

**Affiliations:** Megazyme International Ireland, Bray, County Wicklow Ireland

**Keywords:** *endo*-xylanase, Beechwood xylan, Wheat arabinoxylan, Nelson-Somogyi, 3,5-Dinitrosalicylic acid, Xylo-oligosaccharides, Reducing sugars

## Abstract

The most commonly used method for the measurement of the level of *endo*-xylanase in commercial enzyme preparations is the 3,5-dinitrosalicylic acid (DNS) reducing sugar method with birchwood xylan as substrate. It is well known that with the DNS method, much higher enzyme activity values are obtained than with the Nelson-Somogyi (NS) reducing sugar method. In this paper, we have compared the DNS and NS reducing sugar assays using a range of xylan-type substrates and accurately compared the molar response factors for xylose and a range of xylo-oligosaccharides. Purified beechwood xylan or wheat arabinoxylan is shown to be a suitable replacement for birchwood xylan which is no longer commercially available, and it is clearly demonstrated that the DNS method grossly overestimates *endo*-xylanase activity. Unlike the DNS assay, the NS assay gave the equivalent colour response with equimolar amounts of xylose, xylobiose, xylotriose and xylotetraose demonstrating that it accurately measures the quantity of glycosidic bonds cleaved by the *endo*-xylanase. The authors strongly recommend cessation of the use of the DNS assay for measurement of *endo*-xylanase due to the fact that the values obtained are grossly overestimated due to secondary reactions in colour development.

## Introduction

Xylan is one of the most abundant polysaccharides in nature. It consists of a main chain of β-1,4-linked d-xylopyranosyl residue which is variously substituted by residues of 4-*O*-methyl-d-glucuronic acid, d-glucuronic acid or l-arabinofuranose and in some cases is also esterified by acetyl groups [1]. The xylan main chain is hydrolysed by *endo*-1,4-β-xylanase (*endo*-xylanase) to varying extents based on the nature of the xylanase employed (GH family 10 or GH family 11) [2] and the degree of substitution of the xylan chain with 4-*O*-methyl-d-glucuronic acid, d-glucuronic acid or α-linked l-arabinofuranose [3, 4].

Interest in xylan degradation spans a broad range of industries including the paper pulp industry where *endo*-xylanase has been successfully employed in cellulose pulp bleaching [5], improving the digestion of cereal-based feeds for chickens and pigs [6], improving the nutritional quality and digestibility of ruminant fodder [7] and improving the bread-making quality of wheat flours [8]. More recently, they are finding use in the production of xylosaccharides (XOS) and arabino-xylo-oligosaccharides (AXOS) for use as prebiotics in human nutrition [9].

Numerous methods are available for the measurement of *endo*-xylanase, but many of these suffer from the same problems as parallel methods for the measurement of other *endo*-hydrolases such as α-amylase and *endo*-cellulase (*endo*-1,4-β-glucanase). The first requirement is a sufficiently pure, well-defined substrate. Over the past three decades, the preferred substrate for the measurement of *endo*-xylanase has been the 4-*O*-methyl glucuronoxylan from birchwood, with measurement of enzyme activity by the increase in reducing sugar level as hydrolysis proceeds. In industry, the preferred reducing sugar procedure has been the 3,5-dinitrosalicylic acid (DNS) method of Sumner and Miller [10, 11] as detailed in an interlaboratory study reported by Bailey et al. [12]. Xylose is traditionally used as the standard even though it is well known that the DNS reagent gives a higher colour response with xylo-oligosaccharides than with xylose resulting in overestimation of the xylanase activity [13]. This problem was first noted in attempts to use the DNS assay for measurement of starch hydrolysis by α-amylase [14] and has also been reported in studies on the measurement of *endo*-cellulase using carboxymethyl cellulose as substrate [15]. In the measurement of *endo*-cellulase with the DNS method, cellobiose is routinely used as the standard in an attempt to minimize this overestimation of enzyme activity due to DNS hydrolysis of the cello-oligosaccharides. Activities obtained with the DNS method do not match those obtained with other reducing sugar methods such as the Nelson [16] and Somogyi [17] (NS) arsenomolybdate method.

*endo*-Xylanase is also assayed with soluble chromogenic substrates such as Azo-xylan and Azo-wheat arabinoxylan [18] and insoluble (crosslinked) chromogenic wheat arabinoxylan (AZCL-Wheat Arabinoxylan) available as Xylazyme AX tablets [19]. Such substrates and assay procedures find widespread application in standardizing industrial enzyme preparations as well as measuring trace levels of *endo*-xylanase in plant products and animal feeds. However, such methods must be standardized against the rate of hydrolysis of a native substrate so that release of dyed fragments can be related to the rate of glycosidic bonds cleaved in a native substrate (International Units of enzyme activity; micromoles of bonds cleaved per minute under defined conditions of temperature and pH). Thus, an accurate and reliable primary assay procedure, which accurately measures the rate of hydrolysis of glycosidic bonds in a “xylan-type” substrate, is essential.

Recently, 4-*O*-methyl glucuronoxylan from birchwood became commercially unavailable, creating a demand for alternative xylan substrate sources. Beechwood 4-*O*-methyl glucuronoxylan (glucuronoxylan) as supplied by Carl Roth as a replacement for glucuronoxylan from birchwood is not sufficiently pure for use in reducing sugar-based assay procedures for *endo*-xylanase. In this paper, we report on and compare four substrates for the measurement of *endo*-xylanase, namely glucuronoxylan from birchwood, purified glucuronoxylan from beechwood, arabinoxylan from wheat flour and a partially debranched arabinoxylan from wheat flour (arabinose: xylose = 26:74). The DNS and NS reducing sugar methods are compared as well as the use of various xylo-oligosaccharides as controls to standardize the assay methods.

## Materials and Methods

### Materials

β-Xylanases from *Aspergillus niger* (cat. no. E-XYAN4; UNIPROT Accession no. P55329; GH 11), *Trichoderma viride* (cat. no. E-XYTR1; UNIPROT Accession no. Q9UVF9; GH11), *Cellvibrio mixtus* (cat. no E-XYNBCM; UNIPROT Accession no. O68541; GH10) and *Neocallimastix patriciarum* (cat. no. E-XYLNP; UNIPROT Accession no. P29127; GH11), wheat flour arabinoxylan medium viscosity (WAX; Lot 40601; cat. no. P-WAXYM; Ara/Xyl = 39:61), acid debranched wheat flour arabinoxylan (ADWAX; Lot 140502; cat. no. P-ADWAX26; Ara/Xyl = 26:74), purified beechwood xylan (Lot 141101; cat. no. P-XYLNBE; 4-*O*-methyl glucuronic acid/xylose = 10:90), xylobiose (cat. no. O-XBI), xylotriose (cat. no. O-XTR) and xylotetraose (cat. no. O-XTE) were obtained from Megazyme International. Birchwood and crude beechwood 4-*O*-methyl glucuronoxylans were obtained from Carl Roth GMBH and Co., Karlsruhe, Germany). All other chemicals used were purchased from Sigma Aldrich, Lennox Laboratory Supplies or Lab unlimited (Carl Stuart Group) and were analytical-reagent grade.

### Methods

Xylose and xylo-oligosaccharide standard solutions were prepared by dissolving xylose, xylobiose, xylotriose and xylotetraose in distilled water to a concentration of approximately 0.6 mM (for the NS method) or 2 mM (for the DNS method). Accurate concentrations were then determined using the phenol-sulphuric acid procedure [20] with xylose as standard. For xylo-oligosaccharides, allowance was made for the relative proportion of anhydro-xylose in the particular oligosaccharide. These solutions were then further diluted for use in either the NS or the DNS assays. Standard curves relating the concentration of the various oligosaccharide to absorbance increase with the DNS assay procedure were prepared by adding 0.2 mL of the oligosaccharide (or xylose) over a range of concentrations to 1.8 mL of the xylan or arabinoxylan substrate (10 mg/mL in 100 mM sodium acetate buffer, pH 4.5). Three milliliters of DNS reagent was then added with mixing and colour was developed as described below. Similarly, standard curves relating the concentrations of the oligosaccharides (and xylose) to absorbance increase with the NS reducing sugar procedure were prepared by adding 0.2 mL of oligosaccharide to 0.5 mL of the xylan or arabinoxylan substrate (12.5 mg/mL in 100 mM sodium acetate buffer, pH 4.5). 0.5 mL of NS reagent D was then added and colour was developed as described below.

#### DNSA Reducing Sugar Method

1. *Preparation of reagents*: 10 g of 3,5-dinitrosalicylic acid plus 2 g of phenol, 0.5 g of sodium sulphite and 10 g of sodium hydroxide were dissolved in 800 mL of water. The volume was adjusted to 1 L and the solution stored in a sealed Duran bottle at room temperature (stable for >2 months). Rochelle’s salt solution was prepared by dissolving 80 g of potassium sodium tartrate in 120 mL of water and then adjusting the volume to 200 mL with water. The solution was stored in a sealed Duran bottle at room temperature and is stable for several years.
2. *Preparation of substrate solutions*: 1.0 g of beechwood xylan, birchwood xylan or wheat flour arabinoxylan was added to 90 mL of 100 mM sodium acetate buffer (pH 4.5) and dissolved by stirring at approximately 50 °C for 10 min on a magnetic stirrer hotplate. The volume was adjusted to 100 mL with 100 mM sodium acetate buffer (pH 4.5) and the solution stored in a well-sealed Duran bottle at room temperature. Substrate was also prepared in 100 mM sodium phosphate buffer (pH 6.0) using the same procedure. Two drops of toluene was added to each bottle to prevent microbial contamination.
3. *Preparation of endo-xylanase preparations*: Pure suspensions of *endo*-xylanase in ammonium sulphate (3.2 M) as supplied by Megazyme (see “Materials” section) were centrifuged in a microfuge at 13,000 rpm for 6 min and the supernatant solution removed with a micropipettor and discarded. The enzyme pellet was dissolved in 1 mL of either 100 mM sodium acetate buffer (pH 4.5) containing bovine serum albumin (BSA), (0.5 mg/mL) or 100 mM sodium phosphate buffer (pH 6.0) containing BSA (0.5 mg/mL), depending on the pH optima of the enzyme. This solution was then added to 9 mL of the same buffer and then further diluted in the same buffer to an enzyme concentration suitable for assay and stored on ice between use. *A. niger* and *T. viride endo*-xylanases were dissolved in acetate buffer at pH 4.5 whereas *N. patriciarum* and *C. mixtus* xylanases were dissolved in phosphate buffer at pH 6.0.
4. *Assay procedure*: Multiple aliquots of 1.8 mL of substrate solution in 16 × 120 mm glass test tubes were pre-equilibrated for 5 min at 40 °C. The reaction was initiated by adding 0.2 mL of pre-equilibrated, suitably diluted *endo*-xylanase solution and incubating the tubes at 40 °C. The reaction was terminated after various time intervals by adding 3 mL of DNSA reagent solution with vigorous stirring. Reagent blanks were prepared by adding 3 mL of DNSA reagent to 1.8 mL of substrate solution plus 0.2 mL of the buffer solution as used in the assay, and the tube contents were mixed immediately. Enzyme blanks were prepared by adding 3 mL of DNSA reagent to 1.8 mL of substrate solution plus 0.2 mL of the enzyme solution as used in the assay and the tube contents mixed immediately. The xylose/xylo-oligosaccharide standards were prepared by adding 3 mL of DNSA solution to 1.8 mL substrate solution plus 0.2 mL of xylose or xylo-oligosaccharide standard (0–2 μmoles/0.2 mL). All tubes (reaction, reagent blanks, enzyme blanks and xylose and xylo-oligosaccharide standards) were placed in a boiling water bath and incubated for 15 min. The tubes were removed from the boiling water bath, and 1 mL of 40 % Rochelles salt solution was added immediately and the tube contents mixed immediately on a vortex mixer. The tubes were cooled at room temperature over approximately 15 min, and the contents were then re-mixed. The absorbance of the xylose and xylo-oligosaccharide standards was measured against the reagent blank at 540 nm. Concurrently, the absorbance of the reaction solutions was measured against the enzyme blank at 540 nm. The rate of hydrolysis was calculated as micromoles of xylose reducing sugar equivalent released per minute. One unit of *A. niger endo*-xylanase activity is defined as the amount of enzyme required to release 1 μmole of xylose reducing sugar equivalents per minute from the xylan or arabinoxylan substrate at pH 4.5 and at 40 °C.

#### Nelson-Somogyi Reducing Sugar Method

1. *Preparation of reagents*: Solution A: 25 g of anhydrous sodium carbonate, 25 g of sodium potassium tartrate and 200 g of sodium sulphate were dissolved in 800 mL of distilled water. The solution was then diluted to 1 L with distilled water and stored in a sealed Duran bottle at room temperature. Solution B: 30 g of copper sulphate pentahydrate was dissolved in 200 mL of distilled water containing four drops of concentrated sulphuric acid. The solution was then stored in a sealed Duran bottle at room temperature. Solution C: 50 g of ammonium molybdate was dissolved in 900 mL of distilled water, and 42 mL of concentrated sulphuric acid was added. Six grammes of sodium arsenate heptahydrate was dissolved separately in 50 mL of distilled water, and this was then added to the ammonium molybdate solution. The combined solution was then diluted to 1 L, mixed well and stored in a sealed Duran bottle at room temperature. (Reagents A to C are stable at room temperature for 2 years). Reagent D: 1 mL of reagent B was added to 25 mL of reagent A (stable for ∼7 days at room temperature). Reagent E: just before use, reagent C was diluted fivefold (e.g. 50 to 250 mL) with distilled water, and this was used on the day of preparation.
2. *Preparation of substrate solutions*: 1.25 g of beechwood xylan, birchwood xylan or wheat flour arabinoxylan was added to 90 mL of 100 mM sodium acetate buffer (pH 4.5) and dissolved by stirring at approximately 50 °C for 10 min on a magnetic stirrer hotplate. The volume was adjusted to 100 mL with 100 mM sodium acetate buffer (pH 4.5) and the solution stored in a well-sealed Duran bottle at room temperature. Substrate was also prepared in 100 mM sodium phosphate buffer (pH 6.0) using the same procedure. Two drops of toluene was added to each bottle to prevent microbial contamination.
3. *Assay procedure*: Multiple aliquots of 0.5 mL of substrate solution in 16 × 120 mm glass test tubes were pre-equilibrated for 5 min at 40 °C. The reaction was initiated by adding 0.2 mL of pre-equilibrated, suitably diluted *endo*-xylanase solution, and the tubes were incubated at 40 °C. The reaction was terminated after various time intervals by adding 0.5 mL of Nelson-Somogyi reagent D with vigorous stirring. Reagent blanks were prepared by adding 0.5 mL of Nelson-Somogyi reagent D to 0.5 mL of substrate solution and 0.2 mL of the buffer solution as used in the assay, and the tube contents were mixed immediately. Enzyme blanks were prepared by adding 0.5 mL of Nelson-Somogyi reagent D to 0.5 mL of substrate solution and mixing well. Enzyme solution (0.2 mL) as used in the assay was then added and the tube contents mixed immediately. The xylose/xylo-oligosaccharide standards were prepared by adding 0.5 mL of Nelson-Somogyi reagent D to 0.5 mL of substrate solution plus 0.2 mL of xylose or xylo-oligosaccharide standard (0–0.6 μmoles/0.2 mL) with mixing. All tubes (reaction, reagent blanks, enzyme blanks and xylose and xylo-oligosaccharide standards) were placed in a boiling water bath and incubated for 15 min. The tubes were then removed from the boiling water bath, and 3.0 mL of reagent E was immediately added with mixing on a vortex mixer. The tubes were allowed to stand for 10 min at room temperature and then the contents were mixed again. The absorbance of the reaction tubes were measured against the enzyme blank at 520 nm. The absorbance of the xylose and xylo-oligosaccharide standards was measured against the reagent blank. The rate of hydrolysis was calculated as micromoles of xylose reducing sugar equivalent released per minute. One unit of *endo*-xylanase activity is defined as the amount of enzyme required to release 1 μmole of xylose reducing sugar equivalents per minute from the xylan or arabinoxylan substrate at a pH of 4.5 or 6.0 (depending on the enzyme assayed) and at 40 °C.
4. Determination of Km. Assays were performed as described above except that the concentration of wheat arabinoxylan varied from 2 to 10 mg/mL in the incubation mixture.

## Results

**Fig. 1:**
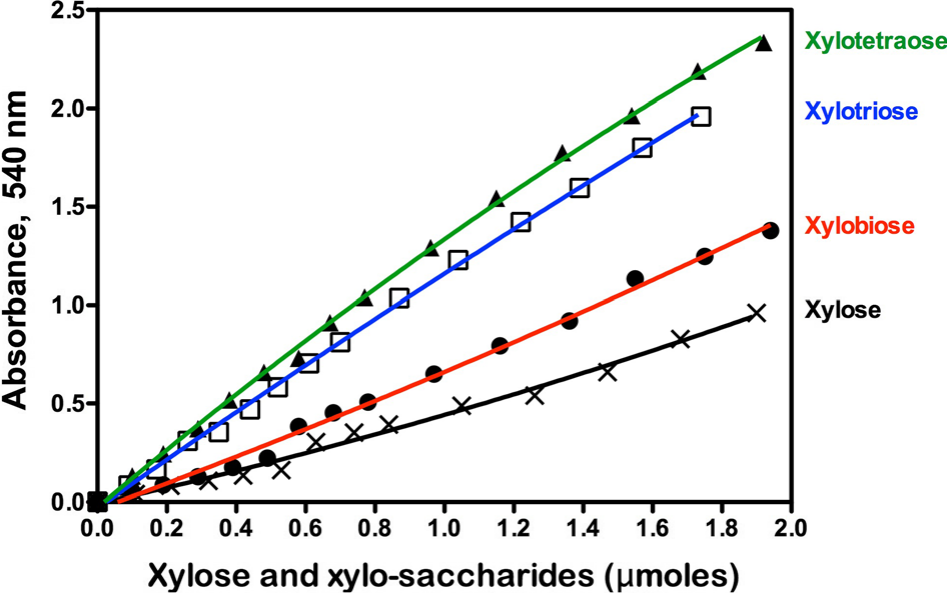
Standard curves for xylose (X), xylobiose (●), xylotriose (■) and xylotetraose (▲) in the presence of beechwood xylan (9 mg/mL) obtained using the DNS reducing sugar method

Standard curves relating the concentration of xylose, xylobiose, xylotriose and xylotetraose (in the presence of beechwood xylan) to the determined absorbance value (540 nm) using the DNS reducing sugar method are shown in Fig. 1. It is evident that the standard curves are not linear over the entire range of 0 to ∼1.8 μmoles of the various sugars. More importantly, the colour responses with equimolar amounts of xylose and xylo-oligosaccharides increase significantly as the degree of polymerization (DP) increases. Over the concentration range of 0.4 to 1.4 μmoles per test, the colour response from xylobiose is 1.32 times that obtained for xylose; for xylotriose, it is 2.28 times higher; and for xylotetraose, it is 2.5 times higher than for xylose. These results are roughly consistent with those previously reported [13] and are in line with observations with malto-oligosaccharides [14] and glucose and cellobiose [15].

**Fig. 2:**
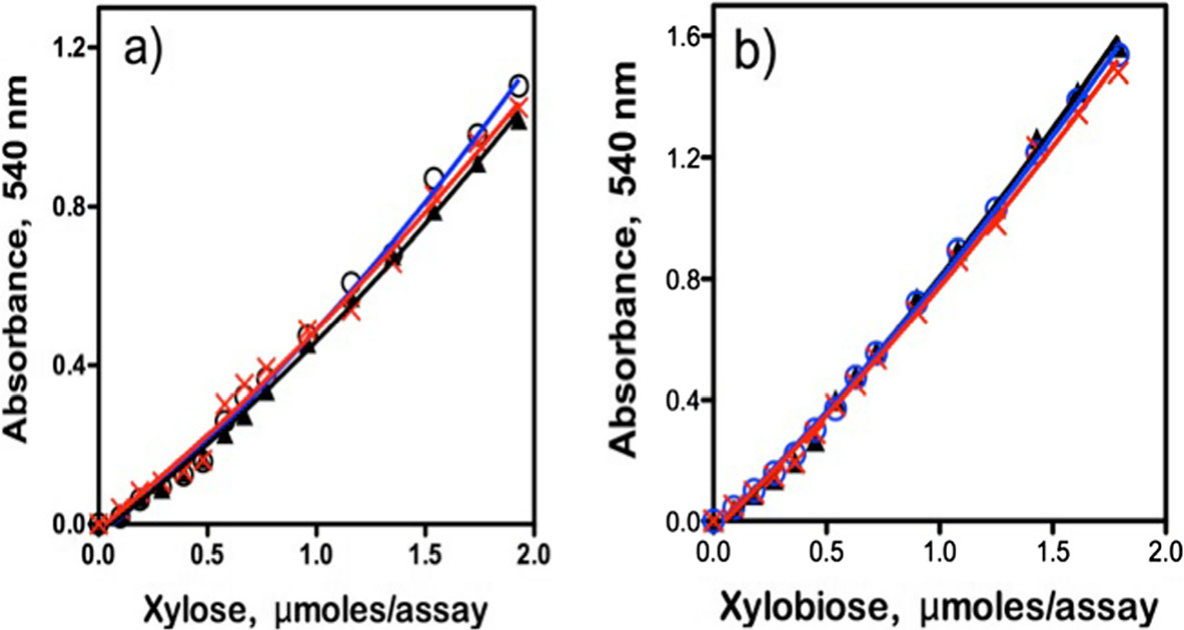
Standard curves for a) xylose and b) xylobiose in the presence of beechwood glucuronoxylan (X), birchwood glucuronoxylan (▲) and wheat flour arabinoxylan () (9 mg/mL) obtained using the DNS reducing sugar method

Since the nature of the xylan substrate used in the procedure for the assay of *endo*-xylanase may affect the colour response obtained from reducing end groups produced during the enzyme reaction, it is important to prepare the xylose (or xylo-oligosaccharide) standard in the presence of the substrate. The effect of beechwood glucuronoxylan, birchwood glucuronoxylan and wheat flour arabinoxylan on the colour response from xylose and xylobiose using the DNS reagent is shown in Fig. 2a, b. Clearly, the standard curves were quite similar in the presence of each of the substrates. The same observations were made for both xylotriose and xylotetraose in the presence of the different substrates.

**Fig. 3:**
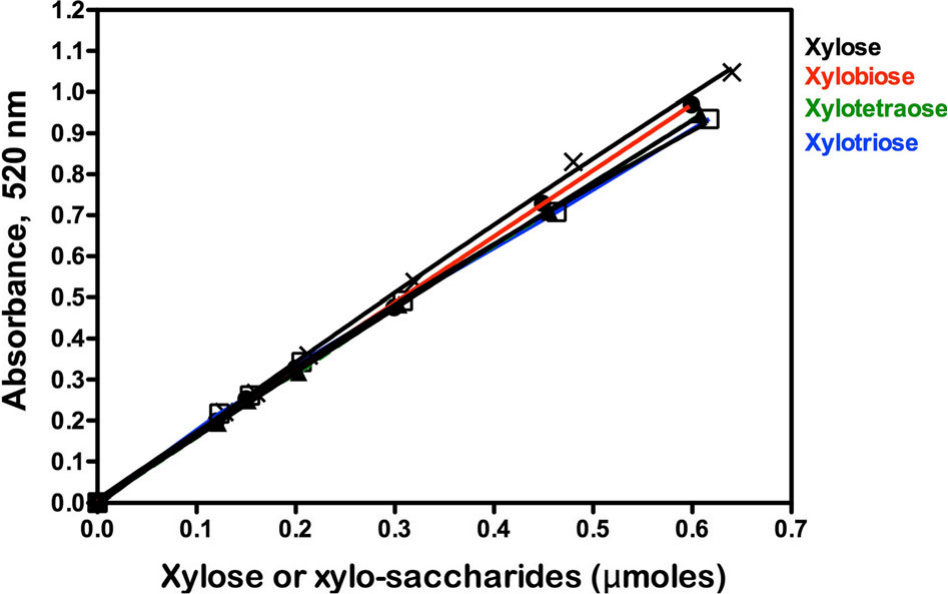
Standard curves for xylose (X), xylobiose (●), xylotriose (□) and xylotetraose (▲) in the presence of beechwood xylan (9 mg/mL) obtained using the NS reducing sugar method

Standard curves relating the concentration of xylose, xylobiose, xylotriose and xylotetraose (in the presence of beechwood xylan) to the determined absorbance value (520 nm) using the NS reducing sugar method are shown in Fig. 3. Clearly, in contrast to the results obtained with the DNS assay, the molar responses from xylose and xylo-oligosaccharides of DP 2–4 are almost identical. This contrasts with the results of Jeffries et al. [13] who reported that the molar response factor for xylobiose was just 65 % of that for xylose and that for xylotriose was 62 %. The reason for this difference is not clear, but may be due to incorrect standardization of the xylo-oligosaccharides used. When the current experiments were performed in the presence of birchwood xylan or wheat arabinoxylan, the same results were obtained, i.e. essentially the same standard curves for xylose, xylobiose, xylotriose and xylotetraose were obtained. These results are in agreement with those reported by Breul and Saddler [15] for glucose and cellobiose using the NS reducing method.

**Fig. 4:**
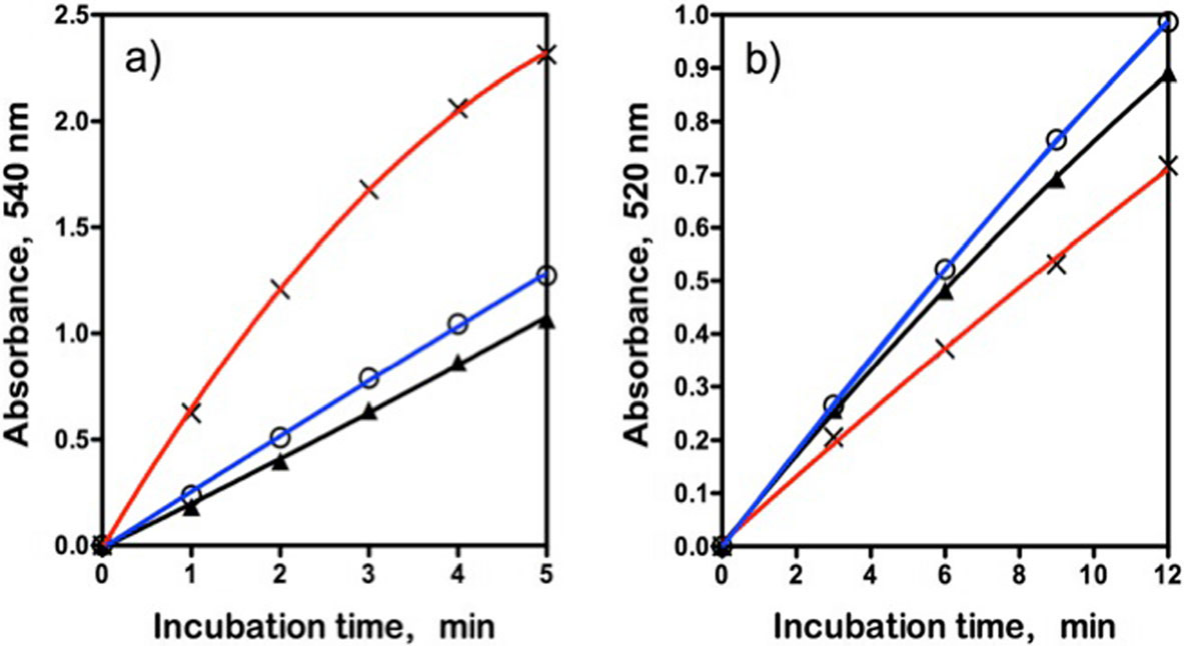
Relative rates of hydrolysis of birchwood glucuronoxylan (▲), beechwood glucuronoxylan () and wheat flour arabinoxylan (X) at 9 mg/mL in the final incubation mixture by *A. niger endo*-xylanase a) determined using the DNS reducing sugar method, and b) determined using the NS reducing sugar method

**Fig. 5:**
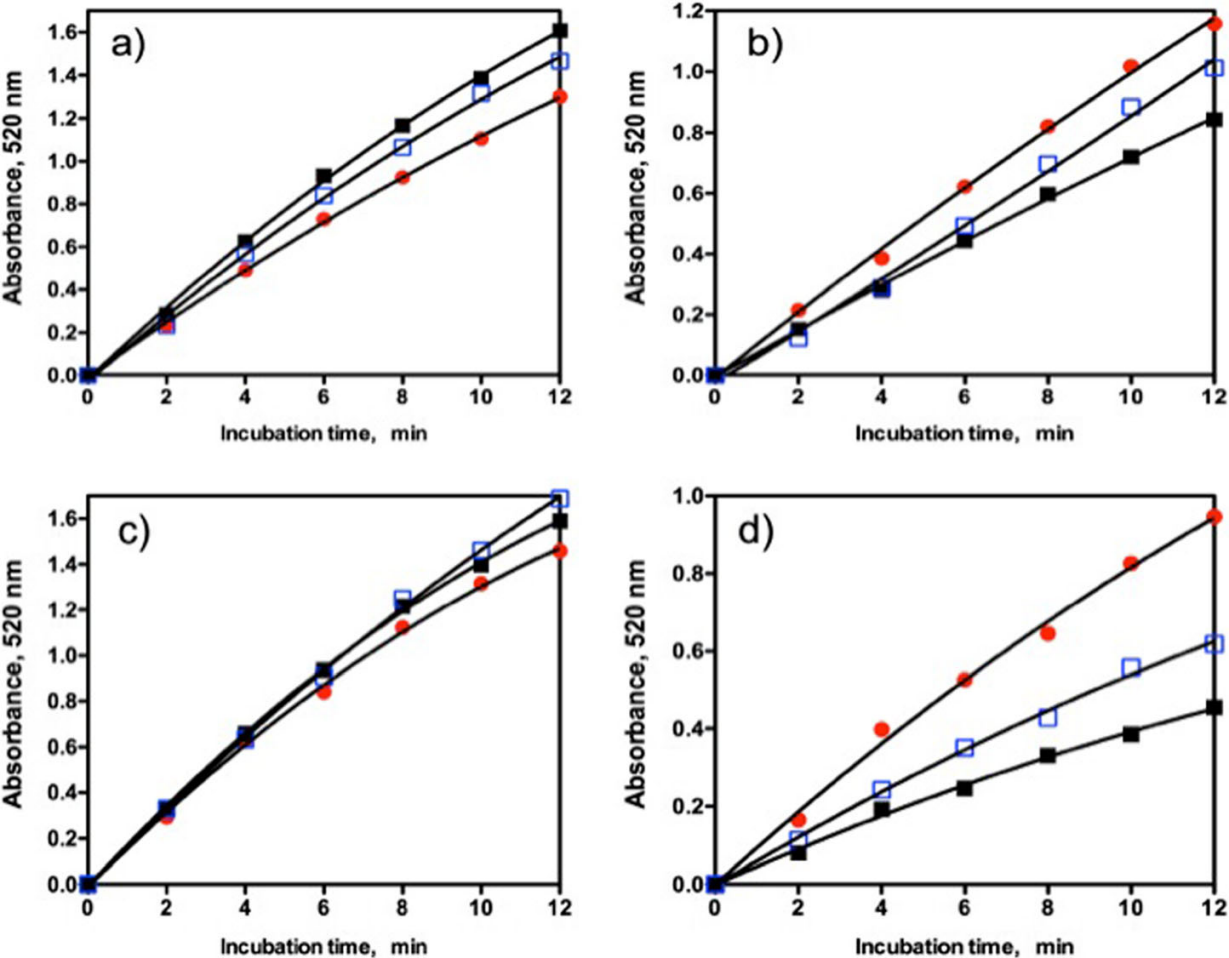
Relative rates of hydrolysis of beechwood glucuronoxylan (), wheat flour arabinoxylan (■) and acid debranched wheat flour arabinoxylan  at 9 mg/mL in the final incubation mixture by a) *T. viride endo*-xylanase, b) *A. niger endo*-xylanase, c) *N. patriciarum endo*-xylanase and d) *C. mixtus endo*-xylanase using the NS reducing sugar method

**Fig. 6:**
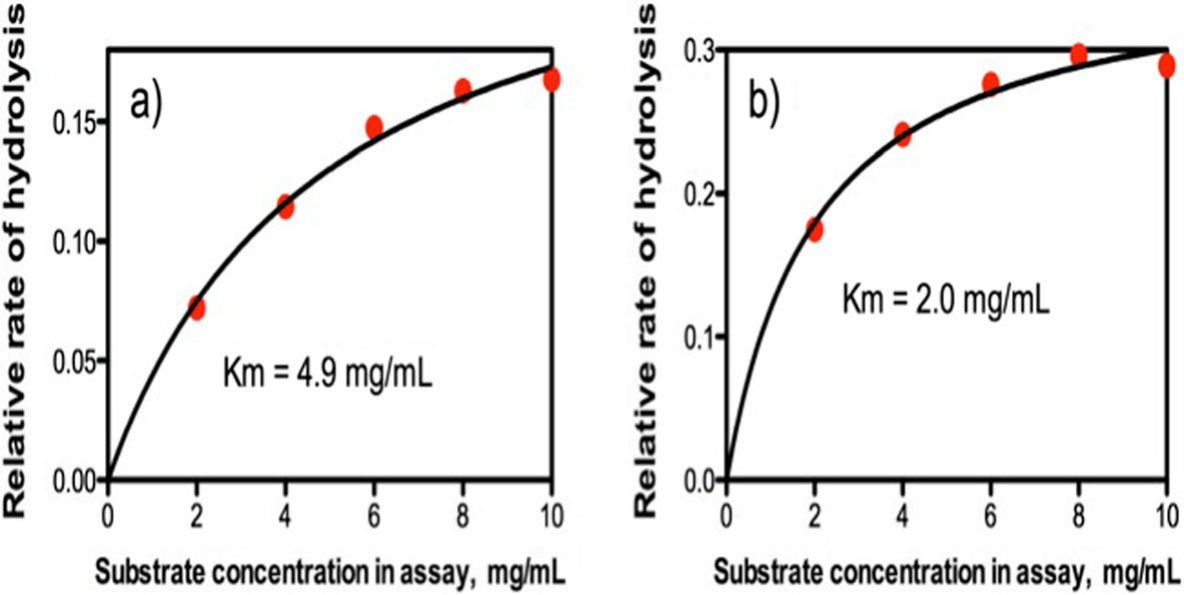
Effect of the substrate concentration on the rate of hydrolysis of wheat flour arabinoxylan by a) *A. niger* and b) *N. patriciarum endo*-xylanases

The relative rates of increase in reducing sugar levels on the hydrolysis of beechwood xylan, birchwood xylan and wheat arabinoxylan by *A. niger endo*-xylanase as determined using the DNS assay are shown in Fig. 4a, and the relative rates as determined using the NS assay are shown in Fig. 4b. Using the DNS assay, similar relative rates of increase in reducing sugar levels were obtained on hydrolysis of beechwood and birchwood glucuronoxylans. This would be expected since both polysaccharides are 4-*O*-methyl glucuronoxylans with a similar content of 4-*O*-methyl glucuronic acid (approximately 10 %). The NS assay also gave similar rates with the two glucuronoxylans (Fig. 4b), but the determined *endo*-xylanase activities were much lower than those obtained with the DNS assay (see Table 1). With the DNS method, a much higher rate of increase in reducing sugar level was obtained with wheat arabinoxylan as substrate compared to beechwood glucuronoxylan. This may reflect a lower level of transglycosylation with the more highly branched arabinoxylan (39 % arabinose content, of which approximately half occurs on singly substituted d-xylosyl residues and half on doubly substituted d-xylosyl residues [21]). Beechwood glucuronoxylan contains just 10 % of 4-*O*-methyl glucuronic acid, meaning that approximately 90 % of the d-xylosyl residues are unsubstituted. In this case, a bulk of the hydrolysis products would be unsubstituted xylosaccharides which can readily serve as acceptors in transglycosylation reactions. With the NS reducing sugar procedure, the apparent rate of hydrolysis of wheat arabinoxylan (increase in reducing sugar level) by *A. niger endo*-xylanase is marginally less than that for the glucuronoxylans. The reasons for this are not clear. The relative rates of hydrolysis of beechwood glucuronoxylan, wheat flour arabinoxylan and acid debranched wheat flour arabinoxylan vary quite significantly between different *endo*-xylanases as shown in Fig. 5. The reasons for the differences cannot easily be attributed directly to the specific action pattern of the enzyme (CAZY family) as quite different relative rates of hydrolysis of the three substrates are observed with endo-xylanases from the same CAZY family, e.g. GH 11 *endo*-xylanases from *A. niger*, *N. patriciarum* and *T. viride*. With *C. mixtus endo*-xylanase (GH 10), significant differences were noted in the relative rates of hydrolysis of the three substrates. This appears to be related to the difference in the degrees of substitution of glucuronoxylan (10 % 4-*O*-methyl glucuronic acid content), wheat flour arabinoxylan (39 % α-linked l-arabinofuranose) and acid debranched wheat flour arabinoxylan (26 % α-l-arabinose). In most cases, acid debranched wheat flour arabinoxylan was hydrolysed slightly more rapidly than wheat flour arabinoxylan, but not as rapidly as beechwood glucuronoxylan (Fig. 5). Clearly, comparison of enzyme activities must involve the use of the same substrate and assay procedure. Substrate concentration is obviously also important, and the effect of the concentration of wheat flour arabinoxylan on the rates of hydrolysis by *A. niger* and *N. patriciarum endo*-xylanases is shown in Fig. 6a, b. The Km for *A. niger endo*-xylanase is 4.9 mg/mL while that for *N. patriciarun endo*-xylanase is much lower at 2.0 mg/mL. The most reliable assay results are obtained if the substrate concentration is approximately twice the Km value. With viscous polysaccharide substrates, this can be difficult to achieve, so in the studies described here, we have settled on a final substrate concentration in the assay mixture of 9 mg/mL.

**Table 1:** Activity determined for a preparation of *A. niger* endo-xylanase using either the DNS or the NS reducing sugar assay with wheat arabinoxylan, birchwood 4-O-methyl glucuronoxylan or beechwood 4-*O*-methyl glucuronoxylan and calculated with xylose, xylobiose, xylotriose or xylotetraose as control

Substrate	Xylose		Xylobiose		Xylotriose		Xylotetraose	
	U/mL	%	U/mL	%	U/mL	%	U/mL	%
(a) DNS assay								
Wheat arabinoxylan	26,760	100	19,020	71	9950	37	8405	31
Birchwood 4-*O*-methyl glucuronoxylan	11,350	100	6595	58	4056	38	3400	29
Beechwood 4-*O*-methyl glucuronoxylan	13,680	100	7750	57	5170	38	4160	30
(b) NS assay								
Wheat arabinoxylan	1470	100	1420	97	1519	103	1516	103
Birchwood 4-*O*-methyl glucuronoxylan	1913	100	1928	101	2029	106	1904	100
Beechwood 4-*O*-methyl glucuronoxylan	1831	100	1751	96	1857	101	1976	108

Values are expressed either as calculated units per milliliter or as a percentage of the value obtained using xylose as the control

The calculated activity of the *A. niger endo*-xylanase on beechwood and birchwood glucuronoxylans and on wheat arabinoxylan, using the DNS and NS methods and xylose, xylobiose, xylotriose or xylotraose as standard, is shown in Table 1. The activity on beechwood and birchwood glucuronoxylans determined with the NS assay and xylose as standard is ∼1900 U/mL independent of whether xylose, xylobiose, xylotriose or xylotetraose are used as standards. With the DNS assay, determined activity values are dependent on which xylosaccharide is used as standard. With xylose as the standard, activity values obtained for *endo*-xylanase on beechwood xylan with the DNS assay are approximately sixfold higher than those obtained with the NS assay procedure. With wheat flour arabinoxylan as substrate, the difference in *endo*-xylanase activity determined with the DNS assay is 18-fold higher than that determined with the NS reducing sugar procedure.

Clearly, the level of *endo*-xylanase activity determined using the DNS assay procedure is grossly overestimated, and this is due to the higher level of colour produced on the reaction of DNS with xylo-oligosaccharides of increasing DP as compared to xylose which is used as the standard in the assay. This effect can be reduced by using either xylobiose or xylotriose as the standard to more closely simulate likely reaction products released on *endo*-xylanase hydrolysis of glucuronoxylan or arabinoxylan. However, even with xylotetraose as standard, the values are still overestimated by twofold. In contrast, the NS reducing sugar assay gives essentially the same colour response with equimolar concentrations of xylose and xylo-oligosaccharides of DP 2–4, indicating that it gives an accurate measure of glycosidic bonds cleaved on hydrolysis of glucuronoxylan (or arabinoxylan) by *endo*-xylanase. The DNS reducing sugar procedure has been widely used for the measurement of *endo*-xylanase following a successful interlaboratory evaluation of this method by Bailey et al. [12]. However, this interlaboratory study only demonstrated that the same incorrect results could be obtained reproducibly. The results obtained have little to do with the number of glycosidic bonds in the glucuronoxylan actually cleaved by the *endo*-xylanase.

## Conclusion

In the studies described here, it has been demonstrated that in the assay of *endo*-xylanase activity, purified beechwood glucuronoxylan is a suitable replacement for birchwood glucuronoxylan, which is no longer commercially available. Alternatively, wheat flour arabinoxylan, also a native substrate, can be employed as substrate. The susceptibility to hydrolysis of beechwood xylan and wheat flour arabinoxylan differs between *endo*-xylanases, meaning that for comparison of enzymes from different sources, the same substrate should be employed. Since the NS assay gives the same colour response with equimolar concentrations of xylose and xylo-oligosaccharides of DP 2–4, it clearly gives an accurate measurement of hydrolytic cleavage by *endo*-xylanase. This is not the case with the DNS reducing sugar method. The NS reducing sugar method is as simple to use as the DNS method, but it needs to be used with care as poisonous chemicals are employed.

Based on the studies above and those by other authors, it is recommended that *endo*-xylanase should be measured with the NS reducing sugar method instead of the inaccurate DNS method. Problems associated with the DNS method have been known for decades, and it is unacceptable that such a deficient method continues to be used. Beechwood glucuronoxylan and wheat flour arabinoxylan are suitable substrates, but the authors recommend the latter as this has been readily available for the past three decades and there is guaranteed supply into the future.

*endo*-Xylanase in fermentation broths, complex enzyme mixtures and food of feed mixtures may occur in admixture with a range of glycosidases, including α-l-arabinofuranosidase and β-xylosidase as well as reducing sugars. This makes specific measurement of the enzyme quite difficult. In such cases, specific and sensitive substrates such as soluble dyed polysaccharides (Azo-xylan and Azo-wheat arabinoxylan) or dyed and crosslinked arabinoxylan (Xylazyme AX tablets) can be employed.
